# ASO Author Reflections: Breast Contour-Preserving Procedures as a Means to Address the Esthetic Outcome of Breast Cancer Treatment

**DOI:** 10.1245/s10434-019-07770-5

**Published:** 2019-09-04

**Authors:** Annelotte van Bommel, Thijs van Dalen

**Affiliations:** 1 Department of Surgery, Leiden University Medical Centre, Leiden, The Netherlands; 2 Dutch Institute for Clinical Auditing, Leiden, The Netherlands; 3 Department of Surgery, Diakonessenhuis Utrecht, Utrecht, The Netherlands

## PAST

The proportion of patients undergoing breast-conserving surgery (BCS) has been used as an outcome parameter for local treatment because BCS is generally considered the preferred local result. Other treatment methods contribute to esthetic outcome as well, either by enabling BCS (e.g., for patients treated with neoadjuvant chemotherapy [NAC]) or by restoring the breast contour in patients undergoing mastectomy through an immediate breast reconstruction (IBR). In addition, the different strategies to maintain the breast shape influence each other. A parameter that comprises the ‘communicating vessels’ for preservation of the breast contour may be more appropriate for evaluation of local esthetic outcome in breast cancer treatment. Breast contour-preserving procedure (BCPP) was defined and explored as a parameter that encompasses the different strategies for preserving the contour of the breast at the time of primary surgery including upfront BCS, BCS after NAC, and mastectomy combined with IBR.

## PRESENT

Data from the Dutch Breast Cancer Audit (NBCA)^1^ of all breast cancer patients in The Netherlands were used for the aforementioned purpose. Between 2011 and 2015, 53% of all patients (*n* = 32,520) underwent upfront BCS. During the study period, the use of NAC and IBR gradually increased respectively from 3 to 8% and 6 to 11% of all patients.^2^ As a result, the overall frequency of BCPP significantly increased from 63% in 2011 to 71% in 2015, whereas the rate of upfront BCS remained unchanged. The BCPP rates were similar for all age categories until the age of 70 years, whereas the means by which the breast contour was preserved varied per age group. The proportion of patients undergoing BCPP ranged extensively between individual hospitals (47–88%), even more than the rate of upfront BCS. Indeed, BCPP provides more comprehensive insight into the way a breast cancer patient can retain her breast contour than the mere rate of BCS.

## FUTURE

Implementation of BCPP in daily practice and combination of this measure with complication rates and data of the perceived esthetic outcome can be used to facilitate shared decision making. The unexplained institutional differences seen for the rate of BCS persist when clinicians adhere to the rate of BCPP as a quality indicator, and this should be a reason for future research. Conversely, knowledge concerning the proportion of patients undergoing BCPP is a more solid base for addressing the large institutional differences with the chance that patients will have a plain mastectomy.
